# Implementing Machine Learning Algorithms to Classify Postures and Forecast Motions When Using a Dynamic Chair

**DOI:** 10.3390/s22010400

**Published:** 2022-01-05

**Authors:** Ghazal Farhani, Yue Zhou, Patrick Danielson, Ana Luisa Trejos

**Affiliations:** 1 Department of Electrical and Computer Engineering, Western University, London, ON N6A 3K7, Canada; gfarhani@uwo.ca; 2 School of Biomedical Engineering (BME), Western University, London, ON N6A 3K7, Canada; yzhou426@uwo.ca; 3 Formid, London, ON N0L 1G0, Canada; patrick@p-dao.com

**Keywords:** dynamic chairs, posture classification, machine learning application, long short-term memory (LSTM), 1D-CNN-LSTM

## Abstract

Many modern jobs require long periods of sitting on a chair that may result in serious health complications. Dynamic chairs are proposed as alternatives to the traditional sitting chairs; however, previous studies have suggested that most users are not aware of their postures and do not take advantage of the increased range of motion offered by the dynamic chairs. Building a system that identifies users’ postures in real time, as well as forecasts the next few postures, can bring awareness to the sitting behavior of each user. In this study, machine learning algorithms have been implemented to automatically classify users’ postures and forecast their next motions. The random forest, gradient decision tree, and support vector machine algorithms were used to classify postures. The evaluation of the trained classifiers indicated that they could successfully identify users’ postures with an accuracy above 90%. The algorithm can provide users with an accurate report of their sitting habits. A 1D-convolutional-LSTM network has also been implemented to forecast users’ future postures based on their previous motions, the model can forecast a user’s motions with high accuracy (97%). The ability of the algorithm to forecast future postures could be used to suggest alternative postures as needed.

## 1. Introduction

Many modern jobs involve prolonged hours of sitting on a chair [1]. It has been shown that prolonged sitting can cause muscle fatigue, poor posture, muscle and joint pain, lower back pain, headaches, and digestive problems [2,3]. Moreover, it can have negative cognitive and psychological effects [4]. Even people with moderate to high physical activity levels can suffer from the side effects of prolonged sitting [5]. For example, it has been shown that after only 15 min of sitting, the mean total intervertebral disc area, lordotic angle, and vertical height of the lumbar spine significantly decrease, which leads to back pain [6]. The lumbar disc pressure is strongly correlated to the sitting postures and level of activity. Hence, developing practical methods to prevent lumbar flattening while seated is an important task [7].

Dynamic chairs, e.g., stability balls, have been proposed to facilitate more active sitting postures, as they allow a person to move while sitting. Not many studies have been conducted to investigate the effect of dynamic chairs on the cognitive and physical health of users; however, the majority of existing studies suggest that changing posture and mixing workstation types can lead to the best outcome [8,9]. The studies also indicate that although the dynamic chairs give the freedom of motion to the users, most people did not take advantage of the increased range of motion, and they often only used a few potential sitting positions. Furthermore, most users were not aware of their sitting habits and body postures [5]. As a solution, recommender systems are being considered, as they have the ability to provide personalized and accurate recommendations to a user [10]. Recommender systems are based on users’ preferences, behaviors, and other additional information. Although they are more common in e-commerce, they have recently been implemented in health services, where they assist in the decision-making process for individuals’ health [11,12]. Similar to the idea of recommender systems, a feedback system can encourage movements, and let the user fully take advantage of the benefits of dynamic chairs. Identifying and forecasting users’ general patterns of motion is an important step toward producing meaningful feedback. Therefore, the focus of the current study is to identify and forecast the general pattern of motion based on sensors located on the chair. This work will provide an important step towards producing meaningful feedback on users’ current and future postures.

In this paper, after reviewing the previous studies, the contribution of the present work will be discussed, and the results of implementing the Machine Learning (ML) algorithms will be shared. Section 2 provides a review of studies of sensorized and smart chairs. A brief discussion on the ML algorithms used for both classification of postures and forecasting next motions is presented. Section 3 provides details on the experimental set-up and postures defined for the training of the ML algorithms. In Section 4, the results of the study are shared. Section 5 provides a discussion related to the results of the study.

## 2. Background

In the literature, several studies have been conducted to identify and analyze users’ seated postures. Some studies proposed the use of wearable sensors [13,14]. However, wearing a sensorized device differs from typical real-life conditions, as when users constantly felt that they were being monitored, their postures would drastically vary from their normal postures. Implementing sensors into chairs is an appropriate alternative that has been used in several studies [15,16,17,18,19,20,21,22].

Different methods have also been proposed to analyze the data from sensors and to relate them to users’ postures. In one of the studies, implementing a fabric pressure sensor array into a cushion was suggested [18]. The cushion was placed on the backrest of an office chair, and it could record pressure in nine different points. Depending on which combination of the nine pressure points had non-zero values, a sitting posture was identified. Unfortunately, the data analysis was limited by the low resolution of the pressure sensors.

More advanced analysis approaches, such as employing ML algorithms, have been proposed as well. For example, in one study, four low-cost load cells were inserted in the seat pad of a chair, and using a Support Vector Machine (SVM) algorithm, they achieved an accuracy of 96% when classifying six different postures. Although the accuracy of the model was promising, load cells required regular and accurate calibrations [19]. In a similar study, 16 pressure sensors (FSR 406, Interlink Electronics; 43.69 mm square sensor, 0.45 mm thick) were inserted into different parts of an office chair. By utilizing ML algorithms, pre-defined postures were successfully classified with the accuracy ranging from 81% to 98% [15]. Finally, the application of more complicated ML algorithms, such as Neural Networks (NNs) has also been studied. Recently a system consisting of six flex sensors was developed, and by using a two-layer NN, seven different sitting postures with an accuracy of 98% were identified [22].

The chairs in the mentioned studies were traditional office chairs; therefore, currently literature does not present classifiers that have been trained to detect fluid motions, as are expected while using a dynamic chair. In dynamic chairs, a user can move with the chair in different directions and postures. For example, they can go forward or to their right and left sides while trying to remain steady on the chair. Furthermore, no study has been found that aims to forecast human posture while using a dynamic chair. Although recommending sitting postures is not within the scope of the current study, being able to forecast future postures could help to suggest alternative postures in the future. To address these gaps, the focus of this paper is to develop ML algorithms capable of identifying a user’s posture, as well as forecasting their future motion when using a dynamic chair.

### 2.1. Machine Learning Methods

ML algorithms have been shown to discover complex underlying functions, and they have been successfully implemented in different fields [23,24,25,26,27]. Recently, many algorithms have been developed for both classification and forecasting tasks. In classification, tree-based algorithms such as Random Forests (RFs) and Gradient Decision Trees (GDTs) are among the most popular, while the SVM algorithm has been proven to be one of the most reliable algorithms for small data sets. In the present study RF, GDT, and SVM algorithms are used for posture identification tasks. These algorithms will be discussed in more detail. Furthermore, as the designed forecasting algorithm is based on a combination of a Long Short-Term Memory (LSTM) and a 1-dimensional Convolutional Neural Network (CNN), these algorithms are also discussed below.

#### Random Forest and Gradient Decision Tree Algorithms

In ML, tree-based algorithms have shown promising results for both classification and regression tasks [28]. An RF is an ensemble of decision trees. Each tree in the forest is grown based on the bagging (bootstrap-aggregation) method, in which samples are drawn randomly with replacements from the original training set. For each node in a tree, a small set of input features are randomly selected for binary partitioning. The splitting criterion is based on choosing a feature, which leads to the lowest Gini Index. The Gini index is defined as $\left(1-\Sigma_{i=1}^{c}P_{i}^{2}\right)$ where $P_{i}$ is the frequency of feature *i* in a specific node, and *c* is the number of classes. In regression, averaging over the output of all the trees is used as the prediction of the RF; while in classification, the corresponding class is selected by taking the majority vote. Details on RFs can be found in [29]. In RFs, the number of trees and the number of features selected per node are two important hyperparameters. When building a tree, it is possible to use bootstrap samples instead of using all of the data set. The option of using bootstrap samples can be considered as a hyperparameter. The complexity of trees including the minimum size of a leaf, the maximum depth of each tree, and the minimum node size to allow new splits should be considered for hyperparameter tuning as well. Typically, the grid search is widely used for hyperparameter tuning, in which an exhaustive search through a manually specified subset of hyperparameters is conducted. Random search is another method used for hyperparameter tuning. In random search, a combination of hyperparameters is randomly selected from the hyperparameter space, such that even less promising choices might be selected. Bergstra [30] showed that by granting random search the same computational capacity as the grid search, it can find better results. After training the model with each of the hyperparameter choices (using either of mentioned methods), the result can be evaluated using the validation set. Finally, the optimal hyperparameters are used in the trained model and for final predictions.

Similar to an RF, a GDT algorithm is also made of trees; however, it is a sequence of simple trees. In GDT, each tree is grown based on the prediction residual of the previous tree with the goal of reducing the new residual. Combining many “weak” learning trees will eventually boost the predictive performance of the algorithm. The final result of the mentioned procedure is a strong classifier. Compared to RF, which is based on training many trees simultaneously, the GDT algorithm is sequential and each tree is grown based on the previous one. A comprehensive description of GDT algorithms can be found in [31]. In GDT the depth of individual trees, as well as the number of features in the terminal nodes, are important hyperparameters.

### 2.2. Support Vector Machine (SVM) Algorithm

Another popular classification algorithm is an SVM algorithm, which is a non-parametric model appropriate for small data sets. An SVM algorithm finds an optimal hyperplane that separates data into classes. If the data are linearly separable, the optimal solution can be directly calculated by maximizing the margin between the separating hyperplane and the data. For non-linear data sets (where a line cannot segment the data set correctly) the feature space is mapped to the higher-dimensional space where the data can be linearly separable. One major success of SVMs is based on using Kernel functions to perform the mapping, so that there is no need to calculate the actual transformation function. One popular choice for the Kernel is the Gaussian Radial Basis Function (RBF), which is used in the current study. Details on the SVMs and Kernel choices can be found in [32]. The coefficients of the non-linear kernels (γ) and the strength of the regularization term (C) are the most important hyperparameters to tune.

The SVMs are designed for binary classification tasks; for multi-class classification, some modifications should be implemented. Error Correcting Output Coding (ECOC) algorithm is a popular classification algorithm based on the theory of error-correcting coding, in which a class is encoded into an *n*-dimensional binary code whose values indicate the class to which a point belongs. In the SVMs, the ECOC can be implemented with the advantage of being able to sort out points in unclassifiable regions [33].

In the current study, the RF, GDT, and SVM algorithms have been implemented to classify users’ postures. Then, the performance of each algorithm was evaluated and compared with the other algorithms.

### 2.3. Long Short-Term Memory (LSTM)

Forecasting a user’s next few postures is the other focus of the present work. A user’s future motion can be predicted based on their previous motions. Thus, a user’s dynamic motion generates a time series. Time series analysis and forecasting have been the topic of many studies [34,35,36]. Machine learning algorithms such as Recurrent Neural Networks (RNNs) (a class of NNs), have recently gained popularity due to their accurate results [37]. RNNs are structured to memorize the information of previous sequences and use them to calculate the current state of a time series. Training in RNN is based on minimizing a loss function that measures the difference between the true target and the output value. The goal of the optimization is to find a weight matrix (*W*) that will produce the optimal estimation of the state vector. The training phase involves forward propagation and backpropagation in time. In forward propagation, each input sequence arrives and is processed through a previous hidden layer, then the estimated value is calculated, and the loss function is computed. The forward propagation continues to get to the end of the training sequence. In the backpropagation step, gradients are backpropagated through layers and through time. One major shortcoming of RNNs is that for long time series, the gradients either explode or vanish. Thus, in practice, RNNs are not useful for longer time series.

A Long Short-Term Memory (LSTM) algorithm is similar to an RNN, but to control the data flow it uses memory blocks consisting of three gates known as input gate, forget gate and the output gate. An LSTM memory block at each time step *t* is defined as follows [38]:

(1) $$i_{t}=\sigma (W_{i}x_{t}+h_{t-1}),$$

(2) $$f_{t}=\sigma (W_{f}x_{t}+h_{t-1}),$$

(3) $$o_{t}=\sigma (W_{o}x_{t}+h_{t-1}),$$

(4) $$c_{t}=f_{t}⊙c_{t-1}+i_{t}⊙\tanh\left(W_{c}x_{t}\right),$$

(5) $$h_{t}=\tanh\left(c_{t}\right)⊙o_{t},$$

where σ is the nonlinear activation function, W is the matrix of network weight parameters to be optimized, $x_{t}$ is the vector of features (time series), $c_{t}$ is known as cell unit at time *t*, $i_{t}$, $f_{t}$, and $o_{t}$ are the input, forget and output gates, respectively, and ⊙ indicates element-wise multiplication. Because of the capability of the LSTM to selectively pass data (through its gates), its backpropagation does not suffer from the explode or vanish gradient problems. Details on the LSTM can be found in [38].

### 2.4. Convolutional Neural Networks (CNNs)

Other promising algorithms in pattern recognition tasks are Convolutional Neural Networks (CNNs). By using a filter (kernel) that moves through the length of the data, a CNN algorithm attempts to only pass the local information (from one layer to the next layer). This means that CNNs significantly reduce the number of features so that they can handle large data sets [39,40]. Traditionally, CNNs were built to handle 2D data such as images and videos. 1D-CNNs are modified versions of 2D-CNNs, which were recently developed to handle 1D signals such as time series [41]. One of the characteristics of the 1D-CNN is that it has one-dimensional kernels. Thus, for time series, the local inter-variable correlation can be extracted [42]. The convolution layer has two parts. The first part performs the convolution operation (using kernels) to extract features, and the second part implements a pooling operation in which features are extracted from the output of the convolution layer. The convolution process for the $l_{th}$ convolution layer is calculated as follows:

(6) $$x_{j}^{l}=f\left(\sum_{i=1}^{N_{l-1}}x_{i}^{l-1}*k_{ij}^{l}+b_{j}^{l}\right)$$

where $x_{i}^{l-1}$ is the input, $k_{ij}^{l}$ is the element of the kernel, and $b_{j}^{l}$ is the bias value. The non-linear function *f* is the activation function operating on the summation of input values multiplied by the kernel weights. Following the convolution layer, the pooling process is implemented to reduce the dimensions of the data. Average pooling in which the average value on the feature map is calculated, and maximum pooling in which the maximum value of the feature map is calculated, are among the most common pooling procedures. The optimization procedure for CNNs is similar to other neural network algorithms, and is based on backpropagation.

Recently, it has been shown that the combination of 1D-CNN and LSTM algorithms can successfully be used for time series forecasting [43,44]. Hence, in the current study, by focusing on each user’s previous postures, a combination of a 1D-CNN and an LSTM (1D-CNN-LSTM) was implemented to forecast their next few postures. Thus, while a user is sitting on a dynamic chair, their current and previous postures are identified and their next postures will be predicted.

## 3. Experimental Setup

### 3.1. Chair Design

**Figure 1:**
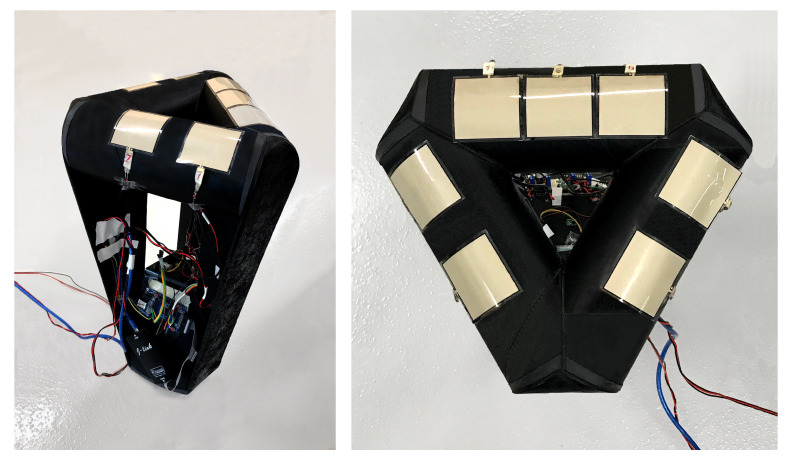
**Left** panel: The view of chair from its side. The accelerometer is attached to the base of the chair. **Right** panel: The view of chair from above: seven FSRs were placed on the top of the chair. The side with three FSRs is the rear orientation.

To develop the proposed ML-based sensorized chair, the Formid dynamic chair was used in this study [45]. The chair has been designed to engage various muscles on the user’s body. Even when a person is sitting in a steady position, due to the geometry of the chair (inverted pyramid with spherical base), constant minor muscle activity is needed to remain steady. The chair was originally designed with an accelerometer in its base, such that the change of movement in three dimensions can be recorded (Figure 1 left panel). As part of this work, seven Force Sensitive Resistors (FSRs) were attached to the seat pan surface, in order to measure the pressure exerted by the body onto the chair in each posture. The cross-section of the chair is very close to an equilateral triangle. The FSRs are equally distanced from each other on each side (Figure 1 right panel).

**Figure 2:**
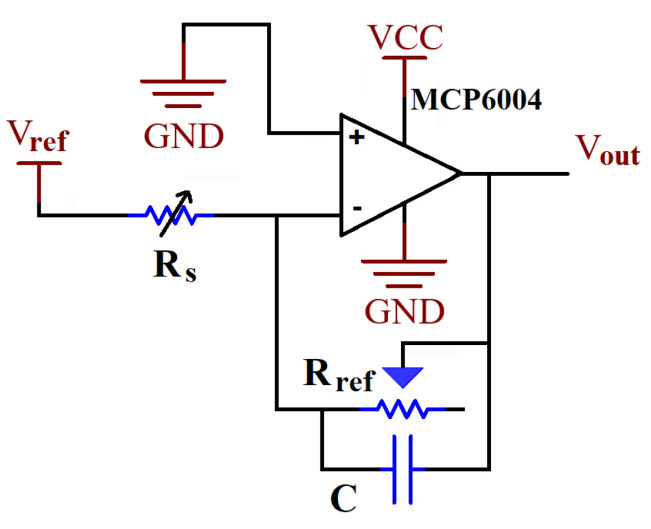
Circuit schematic of the FSR sensors: V${}_{ref}$ is the supplied voltage at −1.1 V, R${}_{ref}$ is a potentiometer of 100 kΩ, R${}_{s}$ is the resistance of the FSR, C is a capacitor of 47 pF, and the MCP6004 is a four-channel low-power operational amplifier.

To measure the exerted force on the chair, the FlexiForce™ A502 FSRs were used, which are standard 50.80 mm square sensors with a 2-pin male connector. The FSRs have a force range of 0 N to 222 N; however, they are only linear through the range of 0 N to 22 N. As recommended by the manufacturer, a circuit was built so that by reducing the resistance of the feedback resistor, linear data for a higher range of applied forces are provided (see Figure 2). The sensors were attached to the chair using a 3M double-sided adhesive tape. To make the chair more comfortable, a padding cushion was tightly attached to the top of the chair. It should be noted that the padding material is not soft, thus the recovery time can be neglected. Furthermore, the experiments contained investigating the static and dynamic postures of a user (detailed discussion is provided in Section 3.2). The time scale of the static part of the experiments was in order of a few seconds, making the time of recovery irrelevant for the analysis. In the dynamic part of the experiments, the movement of the subjects was not changing quickly, therefore, if the firm padding material had any recovery time, it did not have a noticeable impact on the ability to measure the pressure distribution.

**Figure 3:**
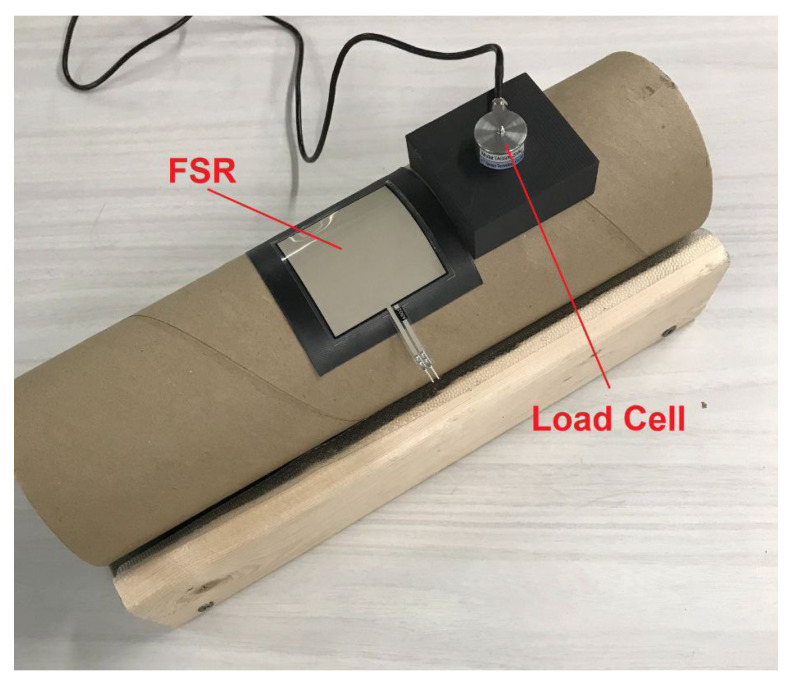
The FSR sensor calibration setup: an FSR was attached to the tube. The tube is made of structural paper which is the same material used to made the chair. When calibrating, the load cell was placed on top of the FSR.

Each FSR was calibrated individually using a TAS606 strain gauge (load cell). A strain gauge measures the change in resistance in response to an applied force and creates an electrical signal proportional to the exerted force. During the calibration process, each FSR was attached to a tube with identical geometry to the triangle side of the chair. The tube is made of structural paper, which is the same material used to make the chair. The load cell was mounted onto a 3D printed attachment that matched the curvature of the FSR and the tube (Figure 3). The direction of the applied force was aligned with the axis of the load cell; therefore, the load was applied and measured when the two surfaces matched, thereby eliminating any concentration of forces on the FSRs and ensuring that the forces acted axially on the load cell. Then, several weights ranging from 1 to 50 kg were used to calibrate the FSRs. Moreover, the hysteresis was assessed by looking at the value reported in the manufacturer document. The document states that the hysteresis is less than 4.5% when applying 80% of the full force, which corresponds to about 1.6 kg in the calibration process. A linear regression equation was fitted to the measured voltage from the FSRs to find the correlation to the applied forces. For all of the FSRs, the R-squared value was higher than 0.98. To evaluate further, the linear model was used to estimate the mass of some weights that were not used for the regression process. The mean absolute percentage error (between the estimated and the actual values) did not exceed 6%.

Seven common sitting postures were identified as follows:

- Neutral seat position, body aligned with gravity (labeled as A).
- Forward seat position, body aligned with gravity (labeled as B1).
- Forward seat position, body aligned with gravity and slouched (labeled as B2).
- Left seat position, body aligned with gravity (labeled as C1).
- Left seat position, body not aligned with gravity (labeled as C2).
- Right seat position, body aligned with gravity (labeled as D1).
- Right seat position, body not aligned with gravity (labeled as D2).

**Figure 4:**
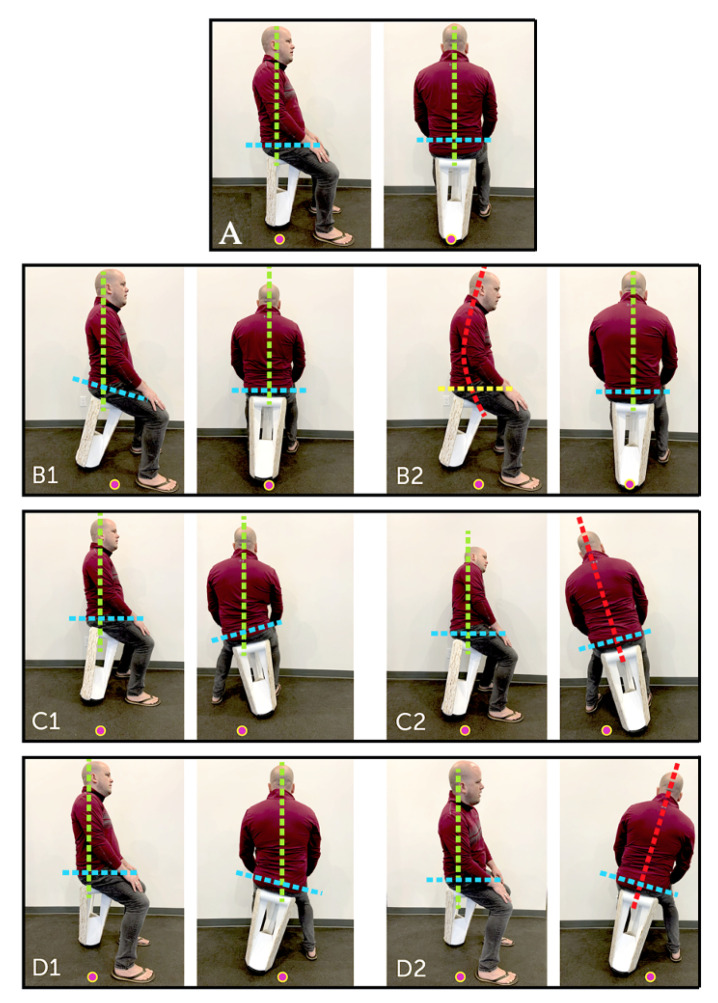
A side view (**left** panel) and rear view (**right** panel) of a variety of postures included in the study. (**A**) Neutral seat position, body aligned with gravity. (**B1**) Forward seat position, body aligned with gravity. (**B2**) Forward seat position, body aligned with gravity and slouched. (**C1**) Left seat position, body aligned with gravity. (**C2**) Left seat position, body not aligned with gravity. (**D1**) Right seat position, body aligned with gravity. (**D2**) Right seat position, body not aligned with gravity. COLOURS: Green: spine aligned with gravity. Red: Spine not aligned with gravity or slouched. Blue: Hips aligned to seat. Yellow: Hip delaminating from seat. Dot: Approximate center of gravity projected down.

Each of the explained postures is shown in Figure 4. These seven postures were used as the target postures for the classification task.

### 3.2. Data Collection

To develop the training set for the classification task, 21 healthy participants (13 women and 8 men) were recruited. As the dynamic chair is designed to engage muscles in the lower body and back, if an individual had a prior muscle injury they would not be recruited. The average height for the women was 166 cm and the average height for the men was 178 cm. The average age of the participants was 29 years old. The recruitment of participants for the study was approved by the Human Research Ethics Board at The University of Western Ontario (Project ID: 117889). Participants were asked to hold each posture for 10 s. Using an LPC1768 microcontroller, data from the sensors were transferred to a laptop through serial communication. After collecting data from their static postures, by providing an environment similar to a work office, participants were asked to sit on the chair for approximately 15 min. While sitting on the chair, the participants were engaged in conversations, working with their laptops, or simply playing with their mobile devices. The period in which the participants were dynamic on the chair was recorded so that their motion could be analyzed and labeled. The collected data from the static postures (pre-defined postures), as well as parts of collected data from the dynamic motion, were used to train an ML classifier.

## 4. Methods

In this section, feature extraction steps, as well as the choice of hyperparameters used for the classification and forecasting algorithms, are provided.

### 4.1. Classifiers

Data were collected from the seven FSRs and the accelerometer attached to the chair, while each participant was holding a posture for 10 s. The quality of the data was checked on site, and if the sensors were disconnected or any other hardware difficulty occurred, the participants were asked to repeat the process. Although the participants were holding a static posture, they had small oscillations and variations that were recorded by the accelerometer and the FSRs. As a reference, the recorded raw data for the *x* direction of the accelerometer are presented in the Supplementary Materials. The mean values of each sensor reading and of the accelerometer were calculated; then the proportion of each FSR value to the total was computed along with the mean value of the accelerometer, and used as the features of the classifier. Furthermore, the participants’ dynamic motions in which they could freely move on the chair were collected. During the trials, the participants’ motions were video recorded, and their postures, with a resolution of 1 second, were labeled manually. Although most participants spent the majority of the time in a neutral posture, three of them were active. Thus, a small portion (less than 5%) of their recorded motion was added to the training data. The recorded sensor values were added to the feature matrix, and the description of the postures was added as labels to the target vector. As mentioned, one common posture in the dynamic motion was a neutral posture accompanied by small-amplitude oscillations. The data associated with the mentioned motion were added to the neutral data set. Another variant was related to the forward motion. Three participants had their legs in front of them while they were holding a forward posture. Moving forward with the chair and leaning on a desk while working with their laptop was observed from two of the subjects, and their data were added into the training data set.

Scikit-learn, which is a free ML library in Python programming language, was used to implement the classifiers. Implementing a Leave One Out Cross-Validation (LOOCV) approach, the classifiers were trained. The codes are available at https://github.com/gfarhani/Posture_classification_Dynamic_chairs (accessed on 20 November 2021). Furthermore, the RandomizedSearchCV module within Scikit-learn was used to perform a randomized search on hyperparameters for the RF. In the RandomizedSearchCV module, the hyperparameters of an estimator are optimized by the cross-validated search over a set of pre-defined options for hyperparameters. The process should be repeated multiple times so that different combinations of parameters are tested.

For the RF hyperparameter tuning, 3-fold cross-validation was used and tested on 500 different combinations. The optimized value for the number of features was found to be $\log(N_{P})$, where $N_{P}$ is the number of features at each node. The optimal maximum depth of each tree was set to be “None,” which means that the nodes were expanded until all leaves were pure (no miss-classification exists in a leaf). The optimal values for the RF hyperparameters are shown in Table 1.

The SVMs and GDTs are much slower algorithms to train. For hyperparameter tuning of these classifiers, a smaller number of parameters combinations (30 combinations) were used. The primary analysis of the SVM results indicated that the data were non-linearly separable. Thus, an RBF kernel was selected as the non-linear choice for kernels. Other possible choices had poor performances. In SVM the optimal performance was observed when C was set to 150, and γ was set to $\frac{1}{N_{P}}$. The GDT algorithm had the highest accuracy when the maximum depth of the trees was set to three and the number of features in the terminal nodes was set to two.

To evaluate the trained classifier, accuracy, precision, and recall were calculated. Accuracy is defined as the proportion of data that are correctly classified. Precision is defined as the proportion of correct predictions among all predictions, and recall is defined as the proportion of correct predictions among all data in one class. The Receiver Operating Characteristics (ROC) curve for each model was plotted as well. ROC is a graphical visualization that illustrates the true-positive rate against the false-positive rate at various threshold settings. The false-positive rate is also known as sensitivity or recall of the classifier, and the false-positive is calculated as 1-recall [46]. The area under the ROC curve (AUC) was calculated as well. AUC shows the aggregated performance of a classifier at different threshold settings and ranges from 0 to 1. If a model classifies all the of labels correctly, it has an AUC of 1, whereas a model that cannot identify any label correctly, has an AUC of 0 [46].

### 4.2. The Forecasting Algorithm

The data set for the subjects’ dynamic motions were segmented into training and test sets. On average, each subject sat on the chair for about 15 min. To train the model, 80% of the data were used while the other 20% were left to test the model (corresponding to the last three minutes of a person’s motions). The model can be trained for each subject based on their previous motions. The algorithm was trained for each of the FSRs and for the *x* and *y* values of the accelerometer. To evaluate the results, the Root Mean Squared Error (RMSE) was calculated:

(7) $$RMSE=\sqrt{\frac{\sum_{i=1}^{N}{\left({\hat{y}}_{i}-y_{i}\right)}^{2}}{N}},$$

where ${\hat{y}}_{i}$ is the predicted value, $y_{i}$ is the ground truth and *N* is the number of points in the test set.

#### 4.2.1. The Architecture of 1D-CNN-LSTM

The network was built using the Keras Application Programming Interface (API) within Python. The general architecture of the network for *x* and *y* consists of one convolutional layer, two LSTM layers, and one fully connected layer. For the FSRs, one convolutional layer, 2 LSTM layers, and two fully connected layers were found as the acceptable base model. The kernel size of the convolutional layer was set to five and the size of the window was set to 50. The LSTM layers contain 200 neurons, and the fully connected layers contain 100 neurons. In all layers, the relu activation function was used. The stochastic gradient descent algorithm was implemented for the optimization process based on implementing the Huber loss function. The Hubler loss function is defined as follows:

(8) $$\left\{\begin{array}{cc} \frac{1}{2}{\left(y-f\left(x\right)\right)}^{2} & |y-f(x)|\leq \delta ,\\ \delta (|y-f\left(x\right)|-\frac{1}{2}\delta ) & otherwise \end{array}\right.$$

where f(x) is the prediction, *y* is the ground truth and δ can be tuned. The Huber loss function approaches the mean squared error loss function when the error (y−f(x)) is small, and it becomes more similar to the mean absolute error function when the error becomes larger. The Huber loss function has the advantage of having high tolerance to outliers [47]. To calculate the optimal learning rate, a sample network was set while its learning rate was gradually changing (after every 20 epochs) from 1e-4 to 1e-8; the learning rate that yielded the smallest loss function was chosen as the optimal learning rate.

#### 4.2.2. Feature Extraction for Forecasting

The forecasting process has two steps. In the first step, the output of each sensor for the next few steps is predicted. In the second step, the output is used as the input of the classifier. For the first step, values for each sensor are individually fed into the 1D-CNN-LSTM algorithm. The data are recorded with the frequency of 20 samples per second. Hence, the average over 20 values is calculated to produce one single value representing one second of motion. Moreover, to reduce noise, a moving average filter with a window size of 5 is used. The output of the filter is fed into the 1D-CNN-LSTM network. The network can forecast the next 32 steps corresponding to the next 32 s of the motion. The result of the forecasting for FSRs and the accelerometer makes a feature vector for the classification model, which in turn will predict a user’s next posture.

## 5. Results

### 5.1. Classification Results and Evaluation

To evaluate the trained models, the accuracy, recall, and precision of each classifier were calculated and shown in Table 2. Moreover, the precision and recall for each label, and for each classifier are shown in Table 3.

**Figure 5:**
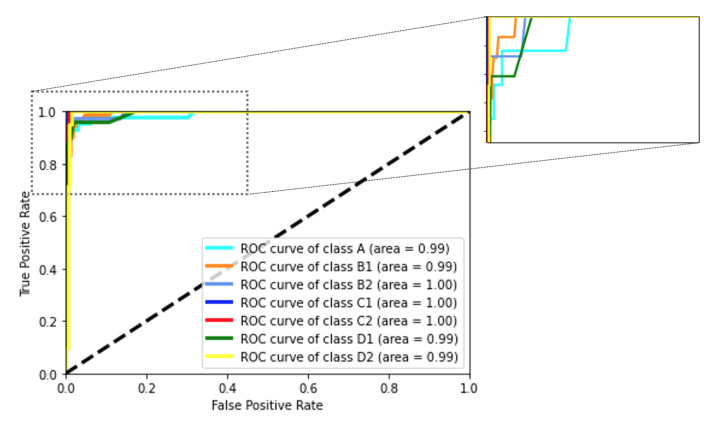
The ROC curve for the RF classifier is shown. The area under the curve for all of the labels is close to 1.0. The upper left part of the plot is zoomed in the inset, to show the variability of thresholds for each class. The classes are consistent with the definition of labels presented in Section 3.1.

**Figure 6:**
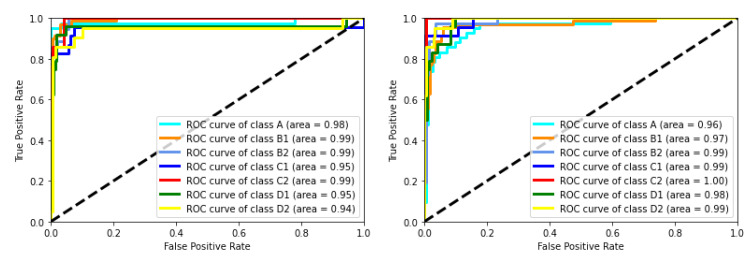
**Left** panel: The ROC curve for the GDT classifier is shown. The area under the curve for all of the labels is higher than 0.90. **Right** panel: The ROC curve for the SVM classifier is shown. The area under the curve for all of the labels is higher than 0.95. The classes are consistent with the definition of labels presented in Section 3.1. The classes are consistent with the definition of labels presented in Section 3.1.

Another metric to evaluate the classifiers is the ROC curve. The ROC curve for the RF classifier is shown in Figure 5. The AUC for all of the labels is close to 1, which indicates the high ability of the classifier to identify different classes. The dashed black line is a reference random classifier. When a curve is on the left side of the dashed line, it means that a classifier works better than a random classifier for that specific label. Similarly, the ROC curves for the GDT and SVM classifiers are shown in Figure 6. The AUC for the GDT algorithm in all classes is higher than 0.90 and for the SVM algorithm is higher than 0.95.

To further investigate if the difference between the models was statistically significant, following the recommendation of [48], a 10-fold cross-validation test followed by a statistical test was performed. In a *k*-fold cross-validation approach, data are randomly divided into *k* disjoint sets of equal-sized sets $T:(T_{1},T_{2},\ldots ,T_{k}$), then a model is trained *k* times. In each trial, an independent test set $T_{i}$, where (i=1,…,k), is evaluated and the accuracy is calculated. In the present work, a 10-fold cross-validation test was performed. Conducting the Shapiro–Wilk test suggested that the distribution of the accuracy scores of the test did not follow a normal distribution. Thus, the Kruskal–Wall test, which is based on investigating the population median of all of the groups, was conducted [49]. The significance level was set to α=0.05. The test showed significant difference among the three methods (p=0.045).

The statistical uncertainty of the classifiers due to the input noise can be used as another factor when evaluating the overall performance of the models and their stability. Using a simple Monte Carlo approach, models were trained 50 times. At each iteration (trial) the input data were perturbed with a random and small Gaussian noise. The reported standard deviation of the Monte Carlo performance was interpreted as the statistical uncertainty of the models [50]. The RF, GDT, and SVM classifiers had statistical uncertainties of 4%, 5%, and 2%, respectively. These small uncertainties due to the input noise indicate that the trained models were robust to the input noise.

The overall reported accuracy of all of the classifiers was high in all cases (above 90%). Moreover, the ROC curve of the classifiers indicates that all of the methods had a high capability of identifying different labels, and had small statistical uncertainty. Considering that the accuracy of all the classifiers is above 90%, in real practice, a model can be trained based on any of these proposed algorithms. However, there exists a statistical difference among the accuracy of the models, where the RF showed slightly better performance. Furthermore, the RF is considerably faster to train. If an on-site retraining of the model is needed, the RF should be selected. For example, it is a great possibility that a user wants to add part of their recent data into the training set and re-train the model. The RF is the fastest model to train, which results in close to real-time predictions. Hence, the rest of this study is focused on RF.

**Figure 7:**
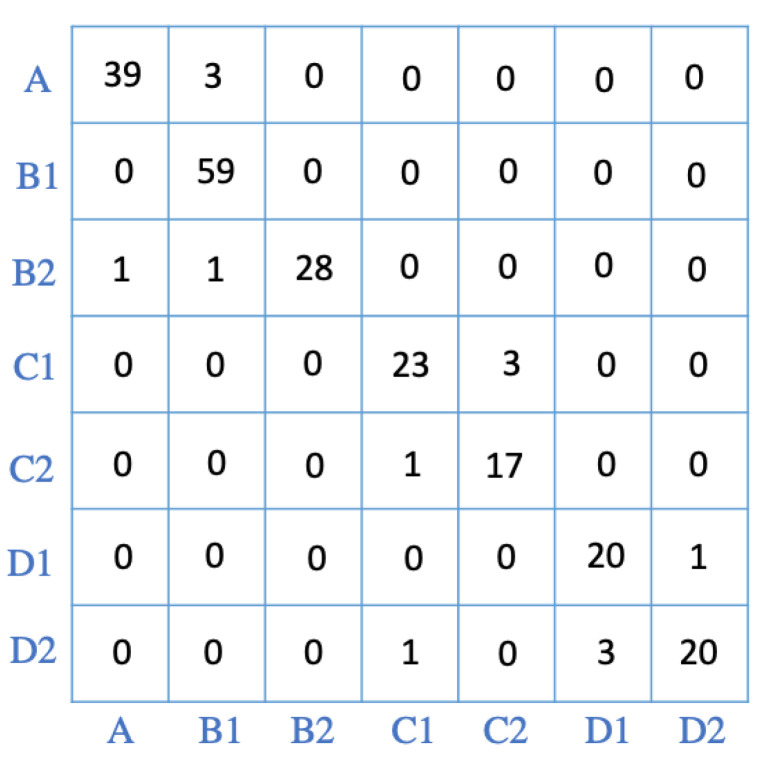
The confusion matrix for the RF model. The classes are consistent with the definition of labels presented in Section 3.1.

As shown in Figure 7, the confusion matrix for RF provides more details on the performance of the algorithm. In a confusion matrix, diagonal values indicate the number of predictions that are correctly labeled in each class, and off-diagonal values show the number of incorrectly labeled cases.

**Figure 8:**
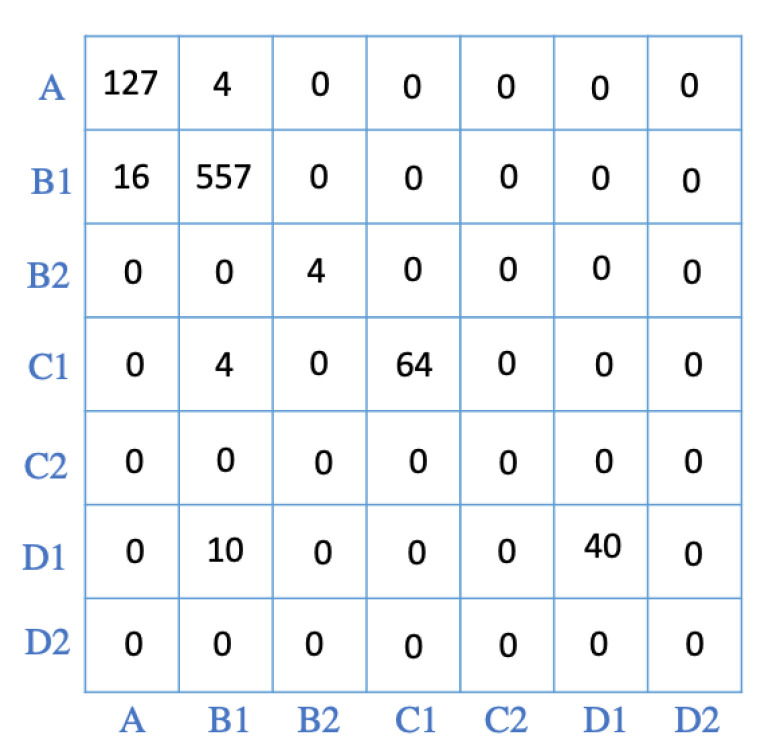
Confusion matrix for 13 min of dynamic motions of a subject. The classes are consistent with the definition of labels presented in Section 3.1.

For further evaluations, the trained model (the RF algorithm) was applied to the unseen data from the subjects’ dynamic motions. The model can be applied to the collected data from each of the 21 subjects. As an example, we have selected about 13 min of dynamic motions of a participant who showed a high range of motions during their trial. The participant was engaged in conversations with people during the trial, or they were talking on their mobile device. Figure 8 shows the confusion matrix for the classification of motions for the mentioned subject. After holding different postures, the participant changed to a neutral posture. They remained neutral for only a few seconds before trying a new posture. The algorithm could correctly label most of the data corresponding to the neutral posture. The classifier has a precision of 96%, and a recall of 88% for the neutral posture. The main habit of the participant was to move forward while their body remained aligned with gravity (B1). The algorithm could correctly label most of this posture, and only in a few instances did it incorrectly label B1 as neutral (A). Recall and precision of 97% for this class indicate that the algorithm can successfully recognize the motion. For a short period of time, the subject moved forward while they were slouching (posture B2), the classifier could correctly (with no miss-classification) classify the posture. The participant also moved to their right and left while their body was aligned with gravity (postures C1 and D1). For C1, precision was 80% and recall was 100% while for D1, the model had a precision of 100% and a recall of 94%. The overall accuracy of the algorithm was 95%, indicating that the model could successfully classify the dynamic motions of the user.

### 5.2. Forecasting Results

**Figure 9:**
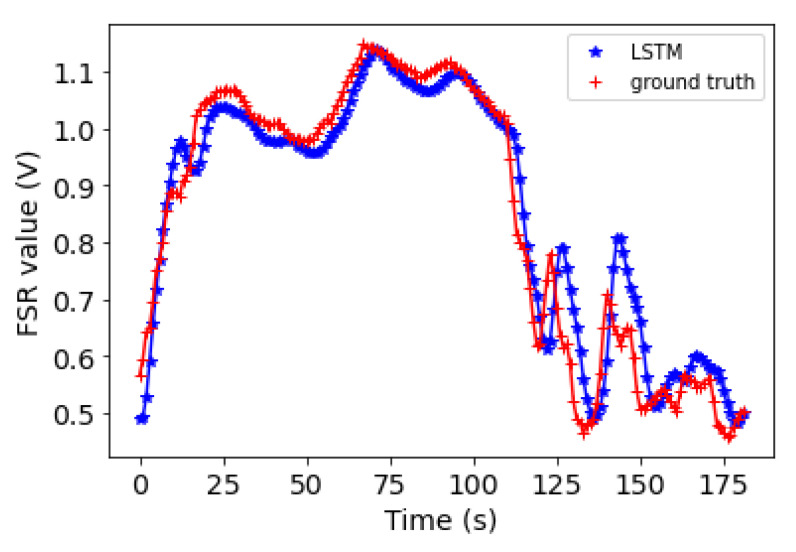
Subject 1: The 1D-CNN-LSTM forecasting values for the next 180 s (blue points) plotted against the actual values (red points) for FSR Number 7.

**Figure 10:**
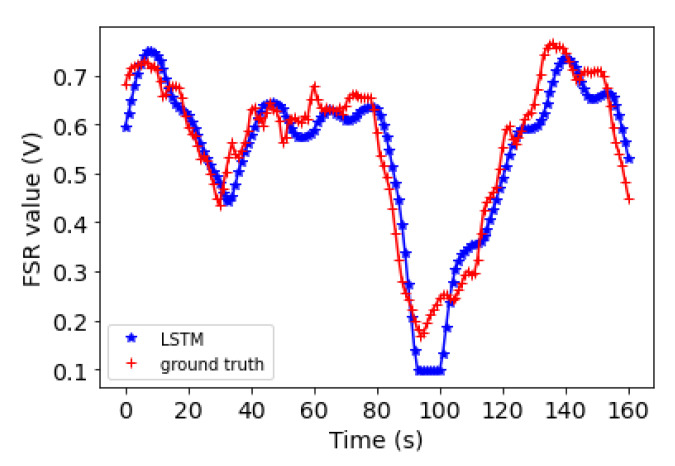
Subject 2: The 1D-CNN-LSTM forecasting values for the next 162 s (blue points) plotted against the actual values (red points) for FSR Number 5.

The performance of the proposed 1D-CNN-LSTM algorithm was also investigated. Table 4 shows the RMSE value for each of the sensors for two of the subjects with high dynamic motions. The RMSE values correspond to the forecasting of the next thirty two steps ahead in time. The average RMSE for Subject 1 is 0.02 and for Subject 2 is 0.06; the result indicates an overall small bias between the ground truth and the predictions. As an example, two plots of forecasting versus the ground truth for one of the FSRs for each of the subjects are shown in Figure 9 and Figure 10.

**Figure 11:**
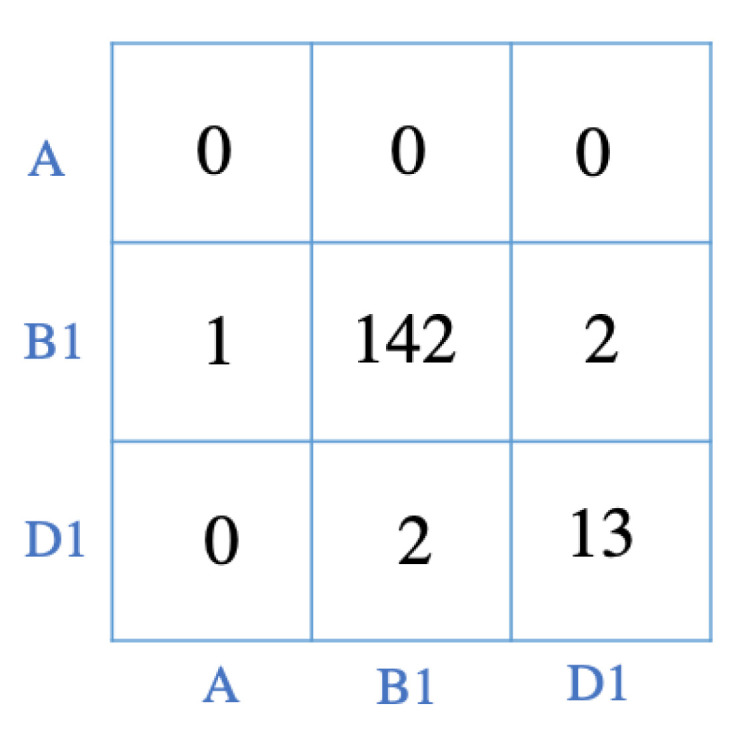
The confusion matrix comparing the forecasting postures with the actual postures. The classes are consistent with the definition of labels presented in Section 3.1.

As a case study, for Subject 2 (whose motion was investigated in the previous section), the results from running the algorithm on the test data set were used as features and fed into the RF classifier. The output of the classifier corresponds to the forecast of the future motions of the user. Moreover, the predicted postures were compared to the actual postures and were shown in the form of a confusion matrix (Figure 11). The forecasting of subject’s motion indicates that they will mostly be in the B1 posture while moving to D1 is predicted as well. Precision for the B1 motion was 97% and recall was 96%. The D1 posture also had precision and recall scores of 87%. The overall accuracy for the forecast was 97%.

## 6. Discussion

ML algorithms were successfully implemented to classify users’ postures on a dynamic chair. The RF, GDT, and SVM classifiers were trained using the LOOCV approach. The RF had overall high accuracy of 94% and was computationally the least expensive model to train. By using the trained RF model, a subject’s postures while they were freely moving on the chair, were classified and compared with the ground truth. The overall accuracy of 95% indicated that the model can successfully identify motions from a new data set. Moreover, the proposed 1D-CNN-LSTM algorithm has the ability to forecast users’ postures for the next 32 steps (32 s). Hence, the algorithm could forecast the next three minutes of a user’s postures based on forecasting the next 32 steps and updating the input of the model with the actual values. The 1D-CNN-LSTM algorithm also has a low RMSE, indicating a low bias between the ground truth and the predictions. In the present work, the discussed examples were associated with two highly active users. However, if a user becomes more static, the model will adjust to the more static behavior as time goes by. The proposed algorithm has comparable accuracy to the algorithms in similar studies of the sensorized chairs discussed in Section 2. However, as the classification task was based on the unique geometry of the chair that allows a user to move freely in different directions, a one-by-one comparison of the results with the previous studies, without having access to their data sets and their experimental setup, was not feasible.

Although the trained classifier has the ability to accurately identify the pre-defined motions of a user, there are many more motions that are not included in the study. Thus, one possible future direction is to train the classifier using more labels, for example, a user can move diagonally or they can move towards the back. One other possible future direction is to use the forecasting results to recommend an alternative posture to a user. For example, if the forecasting algorithm predicts that a user will have a poor posture (based on their previous postures), an alternative can be signaled to them, which has the potential to avoid a sequence of bad habits.

## 7. Conclusions

To summarize, the RF, GDT, and SVM classifiers could identify users’ motions with accuracies of 94%, 91%, and 93%, respectively. The proposed forecasting schema could predict the next 32 motions of a user with an accuracy higher than 95%. Thus, in the present study, the ability of machine learning algorithms to both identify and forecast users’ motions on a sensorized chair was demonstrated.

The algorithms used in the study are easy to implement and can be adapted to other data sets for similar chairs. Identifying users’ postures while they are using the chair can provide them with real-time feedback on their sitting behavior, and potentially alert them of any poor sitting habits. Although the immediate health benefits are not discussed in the paper, the output of the algorithm can potentially be used by professional healthcare providers to recommend alternative postures to each user.

## Figures and Tables

**Table 1:** Optimized values for the RF hyperparameters.

Parameter	Value
Number of trees	10
Number of features selected per node	$\log(N_{P})$
Minimum samples split	5
Minimum sample leaf	1
Maximum depth	None
Bootstrap	True

**Table 2:** Accuracy, precision, and recall for all of the classifiers tested.

Classifier	Accuracy	Precision	Recall
RF	0.94	0.94	0.93
GDT	0.91	0.90	0.87
SVM	0.93	0.93	0.93

**Table 3:** Recall and precision for each class and overall weighted precision and recall of the trained models. Colour: blue columns show result for the RF classifier, green columns show result for the GDT classifier, and yellow columns show result for the SVM classifier. The red row shows the overall weighted average for precision and recall for each classifier.

	RF		GDT		SVM	
Posture	precision	Recall	precision	Recall	precision	Recall
A	0.97	0.93	1.00	0.95	0.95	0.98
B1	0.93	1.00	0.85	0.97	0.99	0.97
B2	1.00	0.92	0.97	0.86	0.95	0.97
C1	0.95	0.87	0.82	0.78	0.96	1.00
C2	0.87	0.95	0.89	0.81	1.00	0.90
D1	0.88	0.96	0.85	0.92	0.84	0.88
D2	0.95	0.86	0.94	0.81	0.85	0.81
Weighted Average	0.94	0.93	0.90	0.87	0.93	0.93

**Table 4:** RMSE values for each sensor for the next 32 steps ahead in time, and for each subject.

Sensor	Subject 1	Subject 2
FSR1	0.013	0.04
FSR2	0.019	0.056
FSR3	0.020	0.057
FSR4	0.007	0.117
FSR5	0.031	0.059
FSR6	0.008	0.076
FSR7	0.062	0.048
*x*	0.004	0.040
*y*	0.017	0.026
Average	0.02	0.06

## Data Availability

The data obtained in this study have not been approved by the Research Ethics Board for open access and are therefore not available to the public.

## Supplementary Materials

The following supporting information can be downloaded at: https://www.mdpi.com/article/10.3390/s22010400/s1, Figure S1: The recorded raw data from the accelerometer for one of the participants while they were holding different postures. Plot a, epresents data for posture A. Plot b, represents data for posture B1. Plot c represents data for posture C1, and Plot d epresents data for posture D1.
