# Supplementary material for: Implementing Machine Learning Algorithms to Classify Postures and Forecast Motions When Using a Dynamic Chair
Source: Sensors (Basel). 2022 Jan 5;22(1):400. doi: 10.3390/s22010400 (PMC8749632; doi:10.3390/s22010400)
Supplement: Supplementary file 1 [file sensors-22-00400-s001.zip › sensors-1509430-supplementary.pdf]

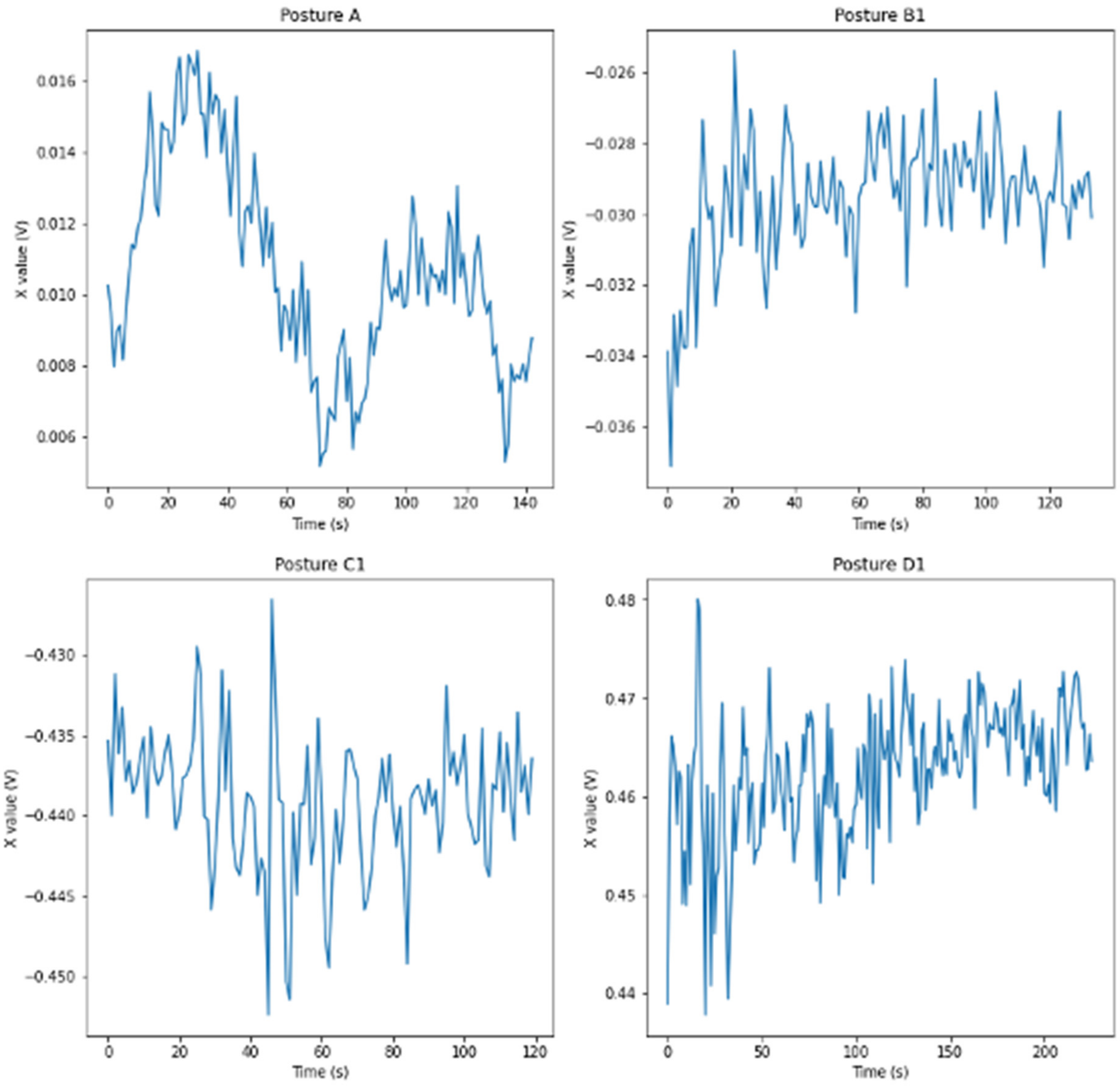

Figure S1: The recorded raw data from the accelerometer for one of the participants while they were holding different postures. Plot a, epresents data for posture A. Plot b, represents data for posture B1. Plot c represents data for posture C1, and Plot d epresents data for posture D1.
